# On Finding and Enumerating Maximal and Maximum *k*-Partite Cliques in *k*-Partite Graphs

**DOI:** 10.3390/a12010023

**Published:** 2019-01-15

**Authors:** Charles A. Phillips, Kai Wang, Erich J. Baker, Jason A. Bubier, Elissa J. Chesler, Michael A. Langston

**Affiliations:** 1 Department of Electrical Engineering and Computer Science, University of Tennessee, Knoxville, TN 37996, USA;; 2 Department of Computer Science, Georgia Southern University, Statesboro, GA 30460, USA;; 3 Computer Science Department, Baylor University, Waco, TX 76798, USA;; 4 The Jackson Laboratory, Bar Harbor, ME 04609, USA;

**Keywords:** graph algorithms, multipartite graphs, maximal cliques, dense subgraph enumeration

## Abstract

Let *k* denote an integer greater than 2, let *G* denote a *k*-partite graph, and let *S* denote the set of all maximal *k*-partite cliques in *G*. Several open questions concerning the computation of *S* are resolved. A straightforward and highly-scalable modification to the classic recursive backtracking approach of Bron and Kerbosch is first described and shown to run in *O*(3^*n*/3^) time. A series of novel graph constructions is then used to prove that this bound is best possible in the sense that it matches an asymptotically tight upper limit on |*S*|. The task of identifying a vertex-maximum element of *S* is also considered and, in contrast with the *k* = 2 case, shown to be *NP*-hard for every *k* ≥ 3. A special class of *k*-partite graphs that arises in the context of functional genomics and other problem domains is studied as well and shown to be more readily solvable via a polynomial-time transformation to bipartite graphs. Applications, limitations, potentials for faster methods, heuristic approaches, and alternate formulations are also addressed.

## 1. Introduction

All graphs we consider are finite, simple and undirected. A graph is *k*-partite if it can be partitioned into *k* nonempty, vertex-disjoint, edgeless subgraphs. The collection of vertices in each such subgraph is called a ‘partite set’. Every edge in a *k*-partite graph is thus ‘interpartite’, that is, it has endpoints in two different partite sets. A *k*-partite graph is complete if it contains all possible interpartite edges. Such a graph is specified by *K*_*x*1_,…,_*xk*_, where *x*_*i*_ denotes the cardinality of partite set *i*, 1 ≤ *i* ≤ *k*.

A *k*-partite clique is a set of vertices that induces a complete *k*-partite subgraph. A *k*-partite clique is maximum if no larger *k*-partite clique exists; it is maximal if no vertex can be added to it to form a larger *k*-partite clique. The quest for maximum and maximal *k*-partite cliques arises in numerous applications, as examples, in textile engineering [1], in categorical data clustering for data mining [2], in the analysis of heterogeneous functional genomics data [3], and in the identification of coherence in protein–protein interaction networks [4].

A *k*-partite graph is balanced if the number of vertices in the various partite sets differ by at most one. Balanced complete *k*-partite graphs are known as Turán graphs, named after Paul Turán, who studied them in the context of extremal graph theory. Turán’s Theorem [5,6] states that such graphs have the maximum number of edges possible for a graph with no (*k* + 1)-clique. Although 2-partite (a.k.a., bipartite) graphs can be recognized in polynomial time, determining whether a graph is *k*-partite for *k* ≥ 3 is *NP*-complete [7]. The complexity is inherited from graph coloring: a graph is *k*-partite if it is *k*-colorable.

Here we derive a number of new results that are highly relevant to this general problem. In the next section, we describe an uncomplicated and efficient technique to enumerate all maximal *k*-partite cliques in a *k*-partite graph. We dub this algorithm MMCE, for maximal multipartite clique enumeration. We base it on the classic backtracking method [8] for finding all maximal cliques in non-partite graphs, and show that it runs in *O*(3^*n*/3^) time. In Section 3, we employ a series of innovative combinatorial constructions to prove an asymptotically tight bound on the maximum number of maximal *k*-partite cliques in a *k*-partite graph, thereby establishing MMCE’s asymptotic optimality. In Section 4, we resolve a significant complexity-theoretic issue by proving that finding a *k*-partite clique with the maximum number of vertices is *NP*-hard for any *k* ≥ 3. Then, in Section 5, we formally characterize a special class of *k*-partite graphs that arises in the context of functional genomics and numerous other application domains. We show such graphs to be more readily solvable via a polynomial-time reduction to bipartite graphs, and devise an *O*(*kn*^3^) time recognition algorithm for them. In a final section, we draw conclusions and offer several directions for future research.

## 2. The MMCE Algorithm

Two different algorithmic variations were proposed by Coen Bron and Joep Kerbosch [8]. One applies a basic backtracking strategy, while a second employs an additional vertex (called a pivot) in an effort to reduce the number of recursive calls required. We will restrict our attention to the more advanced pivot version, which we henceforth term BK. The hallmark of BK is its use of three vertex sets at each recursive call: *R* contains the current clique; *P* contains vertices that can extend the current clique; *X* contains vertices that have already been considered. A major advantage of BK is that maximal cliques do not need to be stored. Instead, they can be discarded as they are discovered. Moreover, duplication of maximal cliques is easily prevented with a clever use of *X*. Only the graph and *R*, *P*, and *X* need be retained in memory. Thus, a signature of efficient BK-based implementations is their low storage overhead.

A litany of BK modifications has been discussed in the literature. Most focus on pivot selection. Such a vertex may, for example, be chosen from *P* ∪ *X* (not just *P*) to minimize the number of recursive calls [9], or it may be selected based on a notion of degeneracy ordering [10]. A more complex alteration has even been developed so that maximal cliques can be enumerated by size [11]. Non-BK approaches have also been proposed [12]. Extensive empirical testing has shown, however, that BK-based algorithms convincingly outperform known alternatives [13].

### 2.1. Multipartite Graphs

We begin by considering the *k* = 2 case. Enumerating bicliques in bipartite graphs has been widely studied. In data mining, for example, the need is often to enumerate closed frequent item sets in transactional data [14]. Faster methods have more recently been devised using a classical graph-theoretical approach [15], with various applications in computational biology [16].

For arbitrary *k*, however, much less seems to have been done. The only known direct method [4] is highly inefficient. It enumerates maximal bicliques for each pair of partite sets. All biclique subsets must then be examined to determine if they can be extended to *k*-partite cliques whenever *k* > 2. As its designers observe, such a brute-force approach scales poorly. A related approach [2] centers on the problem of categorical data clustering, enumerating all subspace clusters. Such a cluster is akin to a *k*-partite clique, but without the requirement that all partite sets be covered. Superficially similar but even more distantly related algorithms include a branch-and-bound strategy [1], improved with the use of bitsets [17]. These methods find only *k*-partite cliques that contain a single vertex from each partite set. They are thus unsuitable as a basis for enumerating maximal *k*-partite cliques.

The following observation is generalized from the well-known relationship between maximal bicliques in bipartite graphs and maximal cliques in general graphs [18].

**Observation 1.** *If G is a k-partite graph, and if a simple graph G′ is built from G by adding all intrapartite edges, then C is a maximal k-partite clique in G if C is a maximal clique in G*^’^ *with at least one vertex in each partite set*.

### 2.2. Algorithm Synthesis

From the foregoing, it naturally follows that any algorithm to enumerate maximal cliques in general graphs can be used to enumerate maximal *k*-partite cliques in *k*-partite graphs. This knowledge forms the genesis of our approach. MMCE (Algorithm 1) first adds intrapartite edges to its input and initializes BK-style vertex sets. It then invokes ENUMERATE, a recursive BK-style subroutine modified to check whether a maximal clique contains a vertex from each partite set. In what follows, we say that a set covers a partition if it contains at least one element from each cell of the partition, and we use *N*(*v*) to denote the neighborhood of vertex *v*, that is, the set of vertices adjacent to *v*.

**Table:** 

Algorithm 1. MMCE
1 input: a *k*-partite graph *G* = (*V*, *E*), with partite sets *V*_*1*_, *V*_2_,. *V*_*k*_;
2 output: all maximal *k*-partite cliques in *G*;
3 add all possible intrapartite edges to *G*;
4 *R* ←∅; *P* ← *V*; *X* ←∅;
5 ENUMERATE (*G*, *R*, *P*, *X*);
6 end MMCE
Subroutine ENUMERATE (*G*, *R*, *P*, *X*)
1 input: a graph *G* = (*V*, *E*), with vertex partition *V*_1_, *V*_2_, …, *V*_*k*_, a clique *R* that covers this partition, and two disjoint subsets *P* and *X* such that *P* ∪ *X* = { *v* ∈ *V*; *R* ⊆ *N*(*v*)};
2 output: all maximal cliques covering this partition that extend *R* with vertices in *P*;
3 if *P* = ∅ and *X* = ∅
4 then if *R* covers the partition *V*_*1*_, *V*_2_, …, *V*_*k*_
5 then report *R* as a maximal *k*-partite clique;
6 return;
7 choose a pivot vertex *u* in *P* ∪ *X* that maximizes |*P* ∩ *N*(*u*)|;
8 for each vertex *v* in *P* \ *N*(*u*)
9 ENUMERATE (*G*, *R* ∪ *v*, *P* ∩ *N*(*v*), *X* ∩ *N*(*v*));
10 *P* ← *P* \ *v*;
11 *X* ← *X* ∪ *v*;
12 end ENUMERATE

**Lemma 1.** *Determining whether a maximal clique contains at least one vertex from each partite set can be accomplished without increasing the overall time complexity of subroutine ENUMERATE*.

**Proof of Lemma 1.** A judicious application of data structures suffices. For example, let us use an array *M* of size *n* to store the partite set membership of each vertex, an array *C* of size *k* to count the number of vertices from each partite set in *R*, and a scalar *t* to record the total number of partite sets currently covered by *R*. *C*(*M*(*v*)) is incremented upon insertion of *v* into *R* (when ENUMERATE is invoked). Similarly, *C*(*M*(*v*)) is decremented upon deletion of *v* from *R* (when ENUMERATE returns). The value of *t* is increased (decreased) iff *C*(*M*(*v*)) goes from 0 to 1 (1 to 0). A single comparison of *t* versus *k* decides whether a maximal clique contains at least one vertex from each partite set. Data structure operations add only a constant number of extra steps to each iteration of ENUMERATE, and thus have no effect on its asymptotic time complexity. □

**Theorem 1.** *The time complexity of MMCE is O(3*^*n/3*^*)*.

**Proof of Theorem 1.** It is known [9,10] that BK runs in *O*(3^*n*/3^) time as long as the pivot is selected as specified on line 5 of subroutine ENUMERATE. No increase results from the addition of intrapartite edges, because this is but an *O*(*n*^2^) task. Nor, thanks to Lemma 1, is an increase incurred by checking whether a maximal clique contains at least one vertex from each partite set. Thus, the time complexity of MMCE is *O*(3^*n*/3^). □

## 3. The Asymptotic Optimality of MMCE

It is well known [19,20] that the maximum number of maximal cliques in a graph with *n* vertices is 3^*n*/3^. The same asymptotic bound holds for the maximum number of maximal bicliques [21]. In the case of bipartite graphs, however, the upper limit on the number of maximal bicliques drops to 2^*n*/2^ [22]. This prompts the following question: how many *k*-partite cliques can reside in a *k*-partite graph when *k* exceeds 2? We now resolve this issue, and in so doing establish that MMCE achieves the best possible asymptotic complexity. Specifically, we will prove that the number of maximal *k*-partite cliques in a *k*-partite graph can essentially be as large as 3^*n*/3^ whenever *k* is at least 3, and thus that any algorithm to enumerate these cliques must take at least Ω(3^*n*/3^) time in the worst case. To make this precise, we employ asymptotic equality, denoted by ~ (tilde), which is defined as follows: *f*(*n*) ~ *g*(*n*) iff $\underset{n\to \infty }{\text{lim}}\left(\frac{f(n)}{g(n)}\right)=1$.

**Theorem 2.** *For each k* ≥ *3, there are infinitely many k-partite graphs for which the number of maximal k-partite cliques is ~3*^*n/3*^.

**Proof of Theorem 2.** An upper bound of 3^*n*/3^ is achieved by combining Observation 2.1 and the aforementioned result for general graphs from [19,20]. Our task thus reduces to showing that this bound is asymptotically tight for an infinite set of *k*-partite graphs whenever *k* exceeds 2. For this, we construct balanced *k*-partite graphs specific to *k* = 3, *k* = 4, *k* = 5, and *k* ≥ 6, each with Ω(3^*n*/3^) maximal *k*-partite cliques.

For the *k* = 3 case, let *n* be evenly divisible by 3, and let *p* = *n*/3. Let *G* result from the removal of the edges of *p* disjoint triangles from a balanced complete tripartite graph of order *n*. More formally, let *G* denote a tripartite graph whose vertices are arranged in partite sets *X*, *Y*, and *Z*, each set of cardinality *p*, with *X* = {*x*_1_, *x*_2_, …, *x*_*p*_}, *Y* = {*y*_1_, *y*_2_, …, *y*_*p*_} and *Z* = {*z*_1_, *z*_2_, …, *z*_*p*_}, and whose edges are {(*x*_*i*_,*y*_*j*_)|*i* 6= *j*} ∪ {(*x*_*i*_,*z*_*k*_)|*i* 6= *k*} ∪ {(*y*_*j*_,*z*_*k*_)|*j* 6= *k*}. Inspired by the technique pioneered in [20], we consider any tripartition {*I*,*J*,*K*} of the set *S* = {1, 2, …, *p*}. *I*, *J* and *K* are therefore nonempty, pairwise disjoint subsets whose union is *S*. Observe that {A = {*x*_*i*_|*i* ∈ *I*}, *B* = {*y*_*j*_|*j* ∈ *J*}, *C* = {*z*_*k*_|*k* ∈ *K*}} denotes a maximal triclique in *G*. We take advantage of this natural bijection between tripartitions of *S* and maximal tricliques in *G*, enumerating the former in order to count the latter. Setting *a* = *|A|* = |I| and *b* = *|B|* = |J|, nonemptiness requires that every tripartition satisfies 1 2264 *a* ≤ *p* − 2 and 1 ≤ *b* ≤ *p* − *a* − 1. For any fixed *a* and *b*, the number of distinct tripartitions is $\left(\begin{array}{l} p\\ a \end{array}\right)\left(\begin{array}{c} p-a\\ b \end{array}\right)$. Thus, the total number of tripartitions of *S* is $\sum_{a=1}^{p-2}\sum_{b=1}^{p-a-1}\left(\begin{array}{c} p\\ a \end{array}\right)\left(\begin{array}{c} p-a\\ b \end{array}\right)$ which, as we show in Appendix A, equals 3^*n*/3^ − 3(2^*n*/3^ − 1). Assigning this function to *f*(*n*) and 3^*n*/3^ to *g*(*n*), it follows that $\underset{n\to \infty }{\text{lim}}\frac{f(n)}{g(n)}=1$, from which we conclude that the number of maximal tricliques in the family of graphs described by *G* is ~3*n*/3.

**Figure 1.**
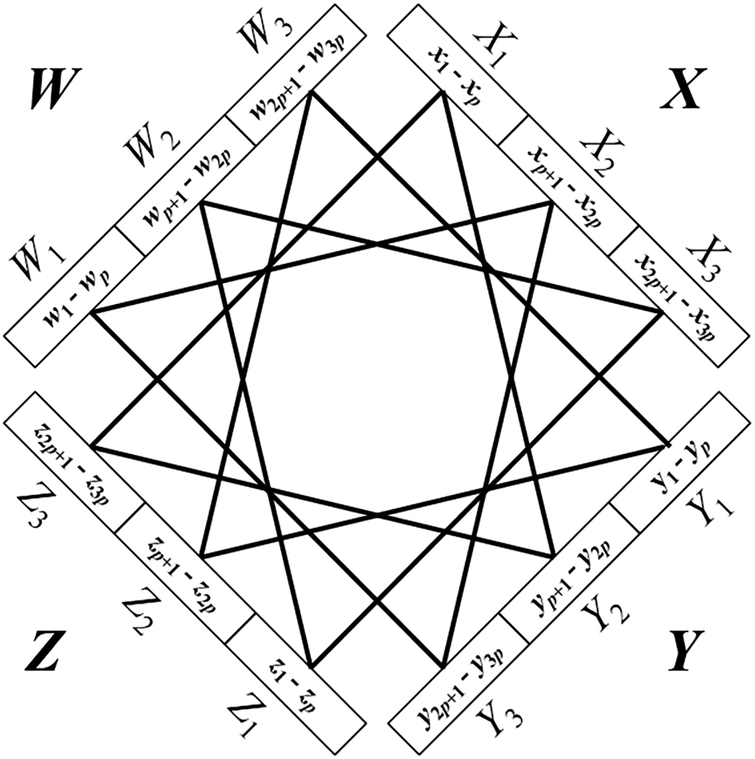
An illustration of the construction used in the *k* = 4 case of Theorem 2. Lines denote bundles of edges that are absent from a balanced complete 4-partite graph of order *n*.

For the *k* = 4 case, let *n* be evenly divisible by 12, and let *p* = *n*/12. We begin by letting *G* denote an edgeless 4-partite graph whose vertices are arranged in partite sets *W*, *X*, Y, and *Z*, each set of cardinality 3*p* = *n*/4, with *W* = {*w*_1_, *w*_2_, …, *w*_3*p*_}, *X* = {*x*_1_, *x*_2_, …, *x*_3*p*_}, *Y* = {*y*_1_, *y*_2_, …, *y*_3*p*_} and *Z* = {*z*_1_, *z*_2_, …, *z*_3*p*_}. For notational convenience, we partition *W* into *W*_1_ = {*w*_1_, *w*_2_, …, *w*_*p*_}, *W*_2_ = {*w*_*p*+1_, *w*_*p*+2_, …, *w*_2*p*_} and *W*_3_ = {*w*_2*p*+1_, *w*_2*p*+2_, …, *w*_3*p*_}, and partition *X*, *Y*, and *Z* in similar fashion. We now place in *G* every interpartite edge except those in the 4*p* disjoint triangles {*w*_*i*_,*x*_*i*+*p*_,*y*_*i*+2*p*_}, {*x*_*i*_,*y*_*i*+*p*_,*z*_*i*+2*p*_}, {*y*_*i*_,*z*_*i*+*p*_,*w*_*i*+2*p*_}, and {*z*_*i*_,*w*_*i*+*p*_,*x*_*i*+2*p*_}, where 1 ≤ *i* ≤ *p*. See Figure 1. Let *M* denote the set of all maximal 4-partite cliques in *G* containing at least one vertex from each of *W*_*i*_, *X*_*i*_, *Y*_*i*_, and *Z*_*i*_, 1 ≤ *i* ≤ 3. Following the reasoning of the *k* = 3 case, there are 3^*n*/12^ − 3(2^*n*/12^ − 1) ways to choose clique candidate vertices from each of {*W*_1_,*X*_2,_*Y*_3_}, {*X*_1_,*Y*_2_,*Z*_3_}, {*Y*_1_,*Z*_2_,*W*_3_}, and {*Z*_1_,*W*_2_,*X*_3_}. Since these four sets do not overlap, we apply the product rule and conclude that *M* contains (3^*n*/12^ − 3(*2*^*n*/12^ − 1))^4^ elements. Assigning this function to *f*(*n*) and 3^*n*/3^ to *g*(*n*), it follows that $\underset{n\to \infty }{\text{lim}}\frac{f(n)}{g(n)}=1$, which we show in Appendix B. Letting *F*(*n*) denote the number of maximal 4-partite cliques in *G*, we have *f*(*n*) ≤ *F*(*n*) ≤ *g*(*n*). By the squeeze theorem of calculus, $\underset{n\to \infty }{\text{lim}}\frac{F(n)}{g(n)}=1$, from which we conclude that the number of maximal 4-partite cliques in the family of graphs described by *G* is ~3^*n*/3^.

**Figure 2.**
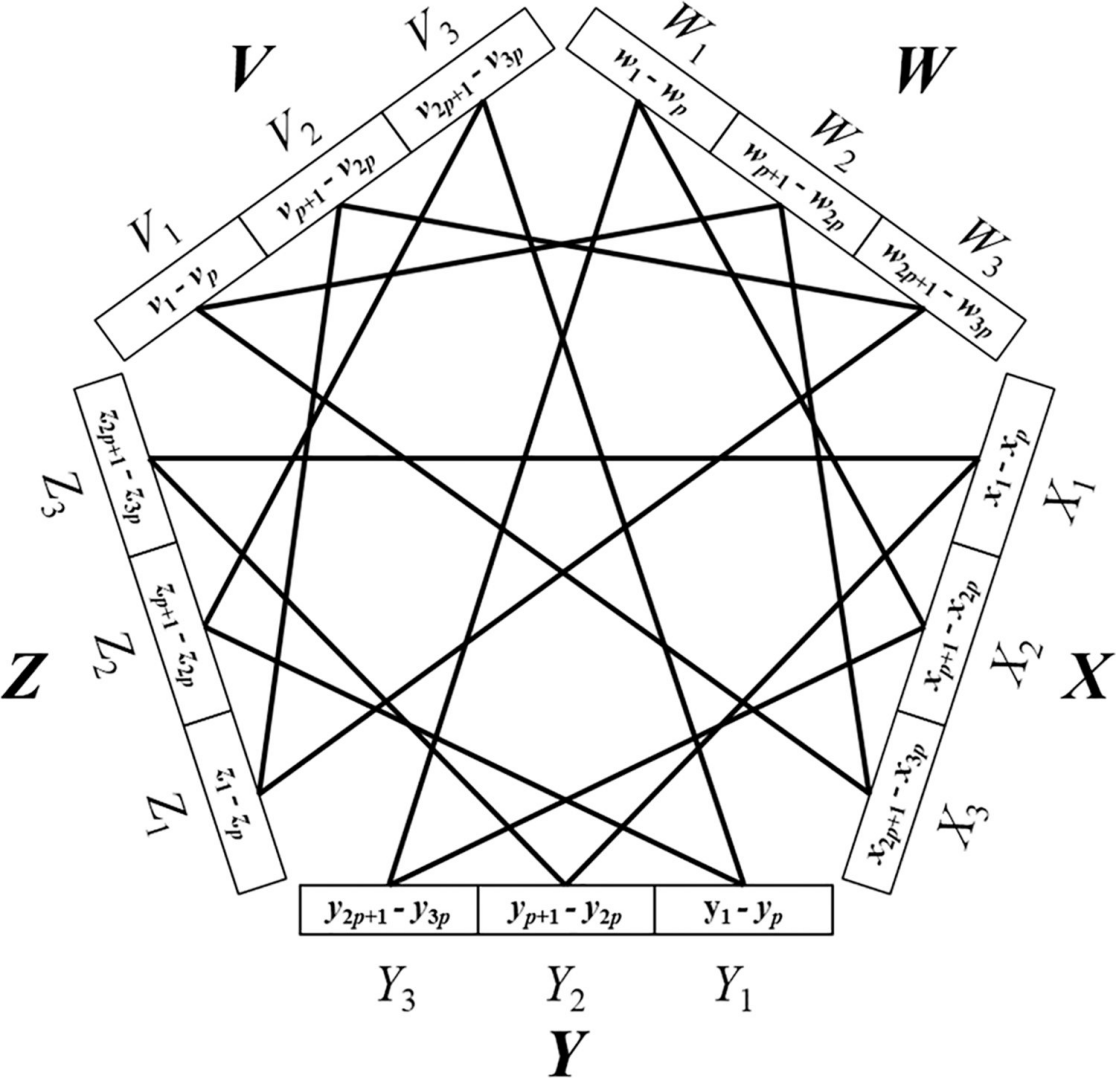
An illustration of the construction used in the *k* = 5 case of Theorem 2. Lines denote bundles of edges that are absent from a balanced complete 5-partite graph of order *n*.

In the *k* = 5 case, let *n* be evenly divisible by 15, and let *p* = *n*/15. We begin by letting *G* denote an edgeless 5-partite graph whose vertices are arranged in partite sets *V*, *W*, *X*, *Y*, and *Z*, each set of cardinality 3*p* = *n*/5, with *V* = {*v*_1_, *v*_2_, …, *v*_3*p*_}, *W* = {*w*_1_, *w*_2_, …, *w*_3*p*_}, *X* = {*x*_1_, *x*_2_, …, *x*_3*p*_}, *Y* = {*y*_1_, *y*_2_,…, *y*_3*p*_} and *Z* = {*z*_1_, *z*_2_, …, *z*_3*p*_}. We partition *V* into *V*_1_ = {*v*_1_, *v*_2_, …, *v*_*p*_}, *V*_2_ = {*v*_*p*+1_, *v*_*p*+2_, …, *v*_2*p*_} and *V*_3_ = {*v*_2*p*+1_, *v*_2*p*+2_, …, *v*_3*p*_}, and partition *W*, *X*, *Y*, and *Z* in similar fashion. We now place in *G* every interpartite edge except those in the 5*p* disjoint triangles {*v*_*i*_,*w*_*i*+*p*_,*x*_*i*+2*p*_}, {*w*_*i*_,*x*_*i*+*p*_,*y*_*i*+2*p*_}, {*x*_*i*_,*y*_*i*+*p*_,*z*_*i*+2*p*_}, {*y*_*i*_,*z*_*i*+*p*_,*v*_*i*+2*p*_}, and {*z*_*i*_,*v*_*i*+*p*_,*w*_*i*+2*p*_}, where 1 ≤ *i* ≤ *p*. See Figure 2. Let *M* denote the set of all maximal 5-partite cliques in *G* containing at least one vertex from each of *V*_*i*_, *W*_*i*_, *X*_*i*_, *Y*_*i*_ and *Z*_*i*_, 1 ≤ *i* ≤ 3. Again following the reasoning of the *k* = 3 case, there are 3^*n*/15^ − 3(2^*n*/15^ − 1) ways to choose clique candidate vertices from each of {*V*_1_,*W*_2_,*X*_3_}, {*W*_1_,*X*_2_,*Y*_3_}, {*X*_1_,*Y*_2_,*Z*_3_}, {*Y*_1_,*Z*_2_,*V*_3_}, and {*Z*_1_,*V*_2_,*W*_3_}. Since these five sets do not overlap, we apply the product rule and conclude that *M* contains (3^*n*/15^ − 3(*2*^*n*/15^ − 1))^5^ elements. Assigning this function to *f*(*n*) and 3^*n*/3^ to *g*(*n*), it follows that $\underset{n\to \infty }{\text{lim}}\frac{f(n)}{g(n)}=1$, which we *n* address in Appendix B. Letting *F*(*n*) denote the number of maximal 5-partite cliques in *G*, we have *f*(*n*) ≤ *F*(*n*) ≤ *g*(*n*). By the squeeze theorem of calculus, $\underset{n\to \infty }{\text{lim}}\frac{F(n)}{g(n)}=1$, from which we conclude that the *n* number of maximal 5-partite cliques in the family of graphs described by *G* is ~3^*n*/3^.

In the *k* ≥ 6 case, we generalize the construction of the last two cases, with *n* evenly divisible by 3*k* and *p* = *n*/3*k*. Let *G* denote an edgeless *k*-partite graph whose vertices are arranged in partite sets *S*_1_*, S*_2_, … *, S*_*k*_, each set of cardinality 3*p* = *n*/*k*, with *S*_1_ = {*s*_1,1_, *s*_1,2_, …, *s*_1,3*p*_}, *S*_2_ = {*s*_2,1_, *s*_2,2_, …, *s*_2,3*p*_}, …, *S*_*k*_ = {*s*_*k*,1_, *s*_*k*,2_, …, *s*_*k*,3*p*_}. For 1 ≤ *i* ≤ *k*, we partition *S*_*i*_ into *S*_*i*,1_ = {*s*_*i*,1_, *s*_*i*,2_, …, *s*_*i*,*p*_}, *S*_*i*_,2 = {*s*_*i*_,_*p*_+1, *s*_*i*_,_*p*_+2, …, *s*_*i*_,2_*p*_} and *S*_*i*_,3 = {*s*_*i*_,2_*p*_+1, *s*_*i*_,2_*p*_+2, …, *s*_*i*_,3_*p*_}. We now place in *G* every interpartite edge except those in the *kp* disjoint triangles {*s*_1_,*i*,*s*_2_,*i*+*p*,*s*_3_,*i*+2*p*}, {*s*_2_,*i*,*s*_3_,*i*+*p*,*s*_4_,*i*+2*p*}, {*s*_3_,*i*,*s*_4_,*i*+*p*,*s*_5_,*i*+2*p*}, …, {*s*_*k*_−2,*i*,*s*_*k*_−1,*i*+*p*,*s*_*k*_,*i*+2*p*}, {*s*_*k*_−1,*i*,*s*_*k*_,*i*+*p*,s_1_,*i*+2*p*}, {*s*_*k*_,*i*,*s*_1_,*i*+*p*,*s*_2_,*i*+2*p*}, where 1 ≤ *i* ≤ *p*. Let *M* denote the set of all maximal *k*-partite cliques in *G* containing at least one vertex from each of *S*_1,*i*_, *S*_2,*i*_, …, *S*_*k*,*i*_, 1 ≤ *i* ≤ 3. Again following the reasoning of the *k* = 3 case, there are 3^*n*/3*k*^ − 3(2^*n*/3*k*^ − 1) ways to choose clique candidate vertices from each of {*S*_1_,1,*S*_2_,2,*S*_3_,3}, {*S*_2_,1,*S*_3_,2,*S*_4_,3}, {*S*_3_,1,*S*_4_,2,*S*_5_,3}, …, {*S*_*k*_−*2*,1,*S*_*k*_−1,2,*S*_*k*_,3}, {*S*_*k*−1,1_,*S*_*k*,2_,*S*_1,3_}, {*S*_*k*,1_,*S*_1,2_,*S*_2,3_}. Since these *k* sets do not overlap, we apply the product rule and conclude that *M* contains (3^*n*/3*k*^ − 3(*2*^*n*/3*k*^ − 1))^*k*^ elements. Assigning this function to *f*(*n*) and 3^*n*/3^ to *g*(*n*), it follows that $\underset{n\to \infty }{\text{lim}}\frac{f(n)}{g(n)}=1$, which we address in Appendix B. Letting *F*(*n*) denote the number of maximal *k*-partite cliques in *G*, we have *f*(*n*) ≤ *F*(*n*) ≤ *g*(*n*). By the squeeze theorem of calculus, $\underset{n\to \infty }{\text{lim}}\frac{F(n)}{g(n)}=1$, from which we conclude that the number of maximal *k*-partite cliques in the family of graphs described by *G* is ~3^*n*/3^.

In summary, for every *k* ≥ 3, we have shown that an infinite set of *k*-partite graphs exists with ~3^*n*/3^ maximal *k*-partite cliques. □

## 4. Complexity-Theoretic Issues

The size of a *k*-partite clique is typically measured by either its number of vertices or its number of edges. Consider, for example, *K*_8,1,1_ versus *K*_3,3,3_. The former has more vertices, while the latter has more edges. Thus, for any *k* ≥ 2, a vertex-maximum *k*-partite clique may or may not also be edge-maximum, and vice versa. The difference between these two superficially similar versions can be significant. In a bipartite graph, for example, one can find a vertex-maximum biclique in polynomial time [23], while finding an edge-maximum biclique is *NP*-hard [24]. Even merely approximating the size of an edge-maximum biclique to within a constant factor turns out to be *NP*-hard as well, assuming the small set expansion hypothesis [25]. This is not unlike the situation for the maximum clique problem on general graphs. There it is possible to approximate the optimum to within a factor of *O*(*n*(loglog*n*)^2^/(log*n*)^3^) [26], while approximating it to within a constant factor is as hard as solving the problem exactly [27] (a polynomial-time approximation would lead to a proof that *P* = *NP*).

We now demonstrate that this rather curious situation does not extend beyond the bipartite case, by showing that finding a vertex-maximum *k*-partite clique in a *k*-partite graph is *NP*-hard for all *k* ≥ 3. We prove this, naturally, by establishing the *NP*-completeness of the decision version of the problem. In some places our proof strengthens, streamlines and makes precise parts of a graph gadget first suggested in [28], where an *L*-reduction was proposed to provide an inapproximability result. Although the original reduction is rather vague but probably sound, reasoning about the quality of its approximation is incorrect. See Appendix C. In contrast, our proof is disentangled from any inapproximability argument, and extends the reduction to all *k* ≥ 3.

**Theorem 3.** *Given a k-partite graph, G, with k* ≥ *3, and a positive integer, p, it is NP-complete to decide whether G has a k-partite clique with p or more vertices*.

**Proof of Theorem 3.** Membership in *NP* is manifest: a proffered solution can be deterministically checked in *O*(*p*^2^) time. To establish *NP*-hardness, we break the analysis into two cases.

In the *k* = 3 case, we reduce an *NP*-complete variant of 3-SAT to 3-partite independent set, and therefore through complementation to 3-partite clique. Given a Boolean expression, *E*, in 3CNF, the one-in-three-SAT problem asks whether *E* has a satisfying truth assignment so that each clause has exactly one true literal. one-in-three-SAT is *NP*-complete even when restricted to expressions with no negated literals [29], which is the version we employ. So let *E* denote an instance of one-in-three-SAT with *m* clauses and no negated literals. Thus, we may write *E* = *C*_1_ ∧ … ∧ *C*_*m*_, where *C*_*i*_ = *l*_*i*,1_ ∨ *l*_*i*,2_ ∨ *l*_*i*,3_ for 1 ≤ *i* ≤ *m*, and where no *l*_*i*,*j*_ is negated. In polynomial time, we shall reduce *E* to an instance *G* of 3-partite independent set so that *E* is one-in-three satisfiable if the 3-partite complement of *G* has a 3-partite clique of size *p* = 4*m*.

**Figure 3.**
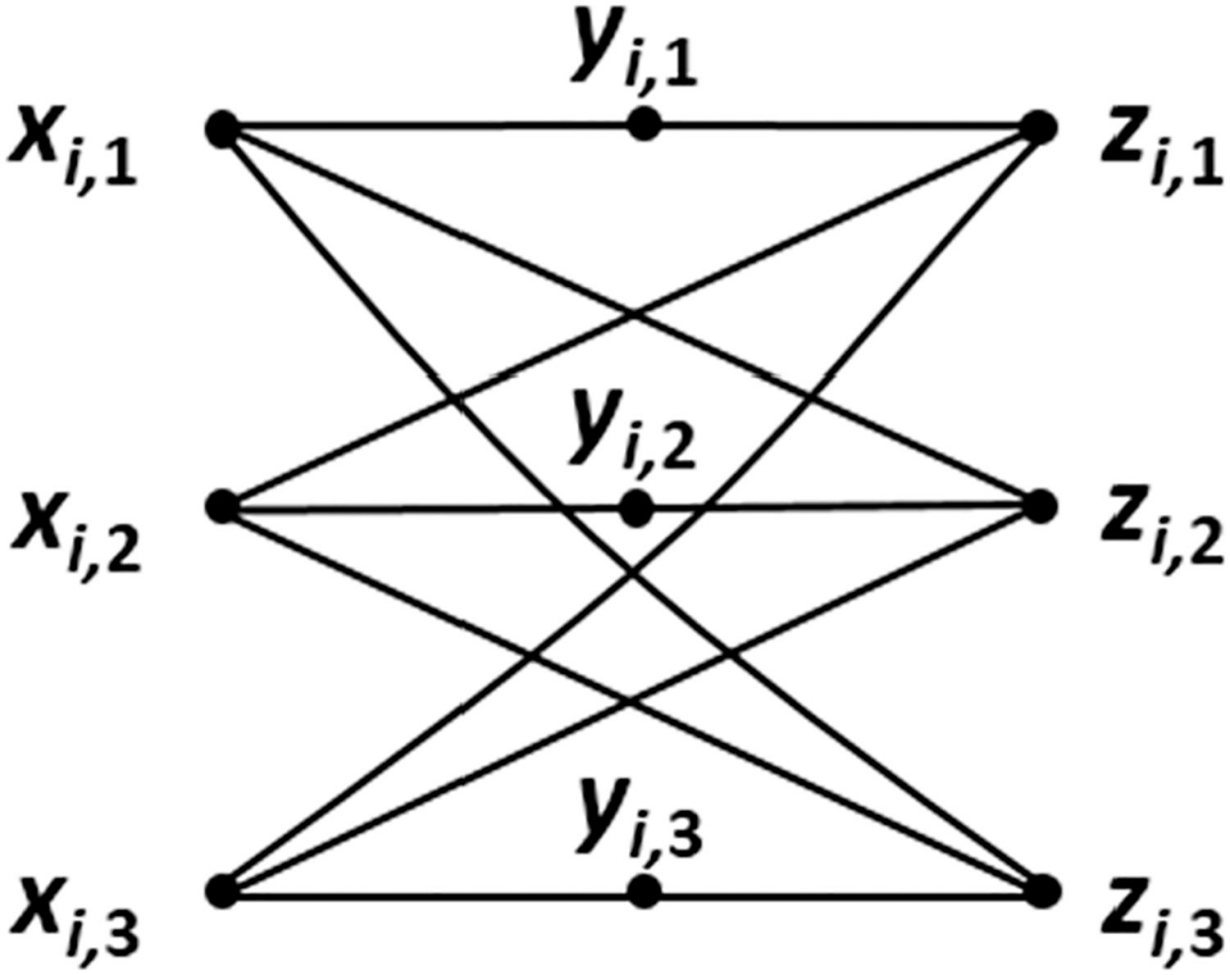
Subgraph produced from the *i*th clause of a one-in-three SAT instance.

**Figure 4.**
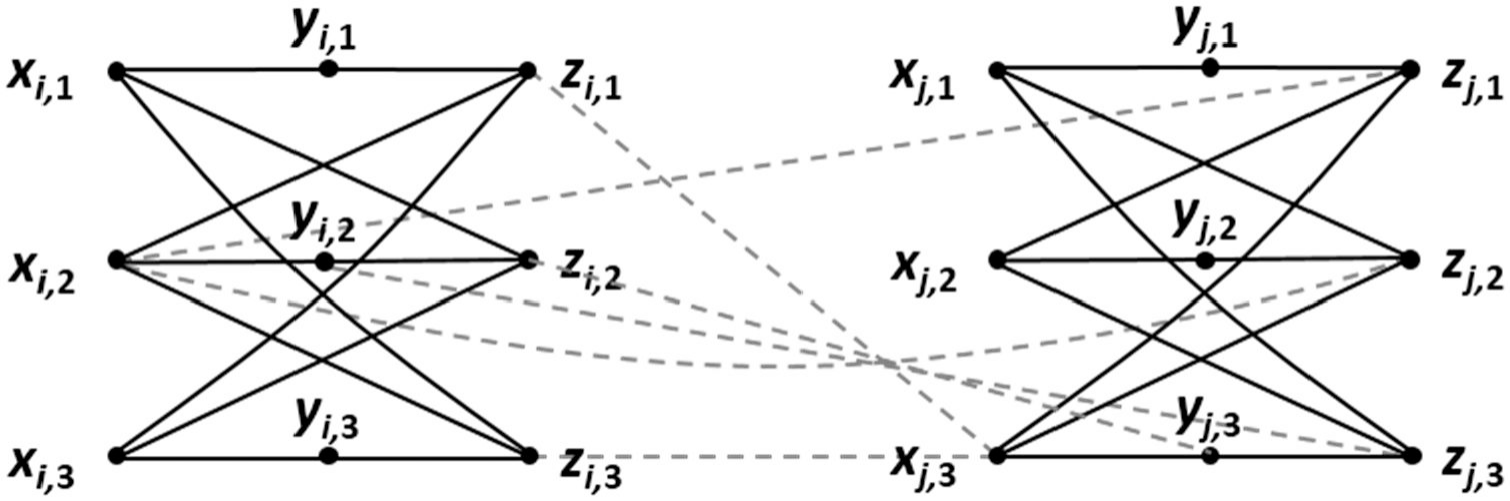
Subgraph connections where *l*_*i*,2_ = *l*_*j*,3_. Dashed lines denote added edges.

**Figure 5.**
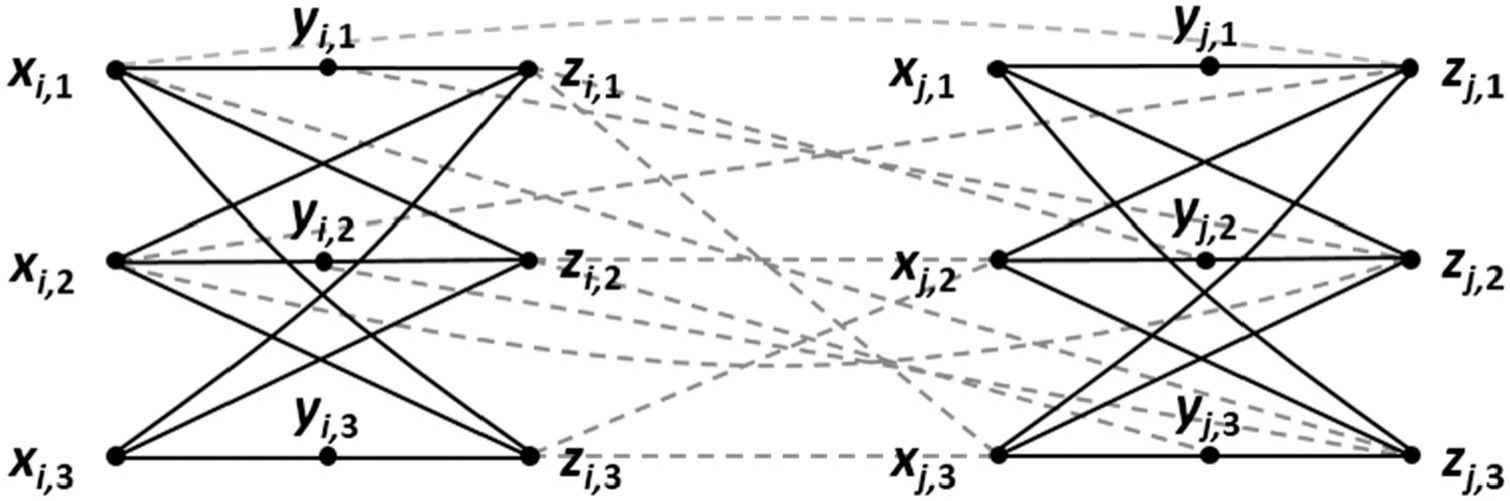
Subgraph connections where *l*_*i*,1_ = *l*_*j*,2_ and *l*_*i*,2_ = *l*_*j*,3_. Dashed lines denote added edges.

The reduction proceeds as follows. *G* contains nine vertices for each clause. Those associated with *Ci* are *xi*,1, *xi*,2, *xi*,3, *yi*,1, *yi*,2, *yi*,3, *zi*,1, *zi*,2, and *zi*,3. To these we add 12 edges: (*xi*,1, *yi*,1), (*xi*,2, *y*_*i*_,2), (*x*_*i*_,3, *y*_*i*_,3), (*x*_*i*_,1, *z*_*i*_,2), (*x*_*i*_,1, *z*_*i*_,3), (*x*_*i*_,2, *z*_*i*_,1), (*x*_*i*_,2, *z*_*i*_,3), (*x*_*i*_,3, *z*_*i*_,1), (*x*_*i*_,3, *z*_*i*_,2),(*y*_*i*_,1, *z*_*i*_,1), (*y*_*i*_,2, *z*_*i*_,2), (*y*_*i*_,3, *z*_*i*,3_). The resulting subgraph is illustrated in Figure 3. Next, we use literals to place edges between subgraphs. Whenever *C*_*i*_ and *C*_*j*_, *i* 6= *j*, contain the same literal *l*_*i*,*s*_ = *l*_*j*,*t*_, we add six more edges: two of the form (*x*_*i*,*s*_, *z*_*j*,*h*_), where *h* 6= *t*; two of the form (*x*_*j*,*t*_, *z*_*i*,*h*_), where *h* 6= *s*; and two of the form (*y*_*i*,*s*_, *z*_*j*,*t*_) and (*y*_*j*,*t*_*, z*_*i*,*s*_). This construction is depicted in Figure 4, using *l*_*i*,2_ = *l*_*j*,3_ as an example. Should *C*_*i*_ and *C*_*j*_, *i* 6= *j*, contain two matching literals, then 12 edges are added. For an example of this see Figure 5, where *l*_*i*,1_ = *l*_*j*,2_ and *l*_*i*,2_ = *l*_*j*,3_. Two clauses cannot of course contain three identical literals and remain distinct. Observe that this transformation creates three independent sets in *G*, namely, those vertices labeled by *x*, those labeled by *y*, and those labeled by *z*. Thus, *G* is a balanced 3-partite graph with 9*m* vertices and 12*m* + 6*d* edges, where *d* is the number of matching literal pairs. The reduction takes at most *O*(*m*^2^) time, realized by comparing each pair of clauses for identical literals. We now show that *E* is satisfiable if *G* possesses an independent set *I* of size 4*m*, with each partite set of *G* containing at least one element of *I*.

We first prove the forward (only if) implication. Suppose *E* is one-in-three satisfiable, and let *A* denote a satisfying truth assignment for *E*. Then consider a candidate for *I* defined by the following rule, where 1 ≤ *i* ≤ *m*: should *l*_*i*,1_ be true under *A*, then *I* contains *x*_*i*,1_, *y*_*i*,2_, *y*_*i*,3_ and *z*_*i*,1_; should *l*_*i*,2_ be true under *A*, then *I* contains *x*_*i*,2_, *y*_*i*,1_, *y*_*i*,3_, and *z*_*i*,2_; and should *l*_*i*,3_ be true under *A*, then *I* contains *x*_*i*,3_, *y*_*i*,1_, *y*_*i*,2_, *z*_*i*,3_. *I* is clearly of size 4*m*, and each partite set of *G* contains at least one (in fact at least *m*) elements from *I*.

It remains only to show that *I* is an independent set. The four vertices that correspond to any single clause are plainly independent. The eight vertices that correspond to a pair of clauses are independent as well, as long as the literals in these clauses are distinct. If a pair of clauses contain any matching literals, however, then there are two possibilities to consider: either the clauses contain just one matching literal or they contain two.

Suppose *C*_*i*_ and *C*_*j*_, *i* ≠ *j*, share exactly one matching literal. Without loss of generality, assume the configuration is as depicted in Figure 4, where *l*_*i*,2_ = *l*_*j*,3_. Should *l*_*i*,2_ (and hence *l*_*j*,3_) be true under *A*, then the aforementioned rule identifies *x*_*i*,2_, *y*_*i*,1_, *y*_*i*,3_, *z*_*i*,2_, *x*_*j*,3_, *y*_*j*,1_, *y*_*j*,2_, and *z*_*j*,3_ as the members of *I*, and indeed these eight vertices are independent. Should *l*_*i*,2_ (and hence *l*_*j*,3_) be false under *A*, then one of the following four events must occur, and again the aforementioned rule applies: *l*_*i*,1_ and *l*_*j*,1_ are true under *A*, and so *x*_*i*,1_, *y*_*i*,2_, *y*_*i*,3_, *z*_*i*,1_, *x*_*j*,1_, *y*_*j*,2_, *y*_*j*,3_, and *z*_*j*,1_ are members of *I*; *l*_*i*,1_ and *l*_*j*,2_ are true under *A*, and so *xi*,1, *yi*,2, *yi*,3, *zi*,1, *xj*,2, *yj*,1, *yj*,3, and *zj*,2 are members of *I*; *li*,3 and *lj*,1 are true under *A*, and so *xi*,3, *yi*,1, *yi*,2, *zi*,3, *xj*,1, *yj*,2, *yj*,3, and *zj*,1 are members of *I*; or *li*,3 and *lj*,2 are true under *A*, and so *xi*,3, *yi*,1, *yi*,2, *z*_*i*,3_, *x*_*j*,2_, *y*_*j*,1_, *y*_*j*,3_, and *z*_*j*,2_ are members of *I*. In each event, *I* contains eight independent vertices.

Now suppose *C*_*i*_ and *C*_*j*_*, i ≠ j*, share two matching literals. Without loss of generality, assume the configuration is as depicted in Figure 5, where *l*_*i*,1_ = *l*_*j*,2_ and *l*_*i*,2_ = *l*_*j*,3_. Should *l*_*i*,1_ (and hence *l*_*j*,2_) be true under *A*, then the aforementioned rule identifies *x*_*i*,1_, *y*_*i*,2_, *y*_*i*,3_, *z*_*i*,1_, *x*_*j*,2_, *y*_*j*,1_, *y*_*j*,3_, and *z*_*j*,2_ as the members of *I*, and indeed these eight vertices are independent. Should *l*_*i*,1_ (and hence *l*_*j*,2_) be false under *A*, then one of the following two events must occur, and again the aforementioned rule applies: *l*_*i*,2_ (and hence *l*_*j*,3_) are true under *A*, and so *x*_*i*,2_, *y*_*i*,1_, *y*_*i*,3_, *z*_*i*,2_, *x*_*j*,3_, *y*_*j*,1_, *y*_*j*,2_, and *z*_*j*,3_ are members of *I*; or *l*_*i*,3_ (and hence, because both pairs of identical literals are false, *l*_*j*,1_) are true under *A*, and so *x*_*i*,3_, *y*_*i*,1_, *y*_*i*,2_, *z*_*i*,3_, *x*_*j*,1_, *y*_*j*,2_, *y*_*j*,3_, and *z*_*j*,1_ are members of *I*. Again, in each event, *I* contains eight independent vertices. We conclude that no matter how pairs of clauses may share matching literals, *I* remains an independent set.

We now prove the reverse (if) implication. Suppose *G* has an independent set *I* of size 4*m*, with the property that each partite set of *G* contains at least one element from *I*. Then consider a candidate truth assignment *A* defined by the following rule, where 1 ≤ *i* ≤ *m* and 1 ≤ *j* ≤ 3: literal *l*_*i*,*j*_ is set to true iff *x*_*i*,*j*_ is in *I*. Our task is to show that *E* is one-in-three satisfied by *A*.

As can be seen from Figure 3, the maximum size of an independent set in the nine-vertex subgraph produced from the *i*th clause of *E* has size four, as realized either by *x*_*i*,1_, *y*_*i*,2_, *y*_*i*,3_, and *z*_*i*,1_, or by *x*_*i*,2_, *y*_*i*,1_, *y*_*i*,3_ a nd *z*_*i*,2_, or by *x*_*i*,3_, *y*_*i*,1_, *y*_*i*,2_, and *z*_*i*,3_. Thus, each such subgraph must contribute exactly four vertices to *I*, each partite set of *G* must contain at least one (in fact at least *m*) elements from *I*, and only one element of *I* can be labeled by *x* in each subgraph. *A* is therefore a one-in-three truth assignment for *E*, and *A* is valid as long as it contains no conflict (a literal set to true in one clause and false in another).

Let us consider then the effect of a conflict between identical literals, *l*_*i*,*s*_ and *l*_*j*,*t*_, where *i ≠ j*. Without loss of generality, suppose *l*_*i*,*s*_ is assigned true and *l*_*j*,*t*_ is assigned false under *A*. This of course means that *x*_*i*,*s*_ is in *I* and *x*_*j*,*t*_ is not. Some *x*_*j*,*r*_, where *r* 6= *t*, must therefore be in *I*. By construction, *z*_*j*,*r*_ is now both in *I* and adjacent to *x*_*i*,*s*_, which is impossible. From this, we conclude that *A* can have no conflicts, and that it constitutes a valid one-in-three satisfying truth assignment for *E*.

We have thus reduced one-in-three-SAT with no negated literals to 3-partite independent set with at least one vertex in each partite set. The reduction to 3-partite clique is completed by complementing only interpartite edges. It follows that *E* is one-in-three satisfiable if the 3-partite complement of *G* has a 3-partite clique of size *p* = 4*m*.

In the *k* > 3 case, extending the reduction to larger values of *k* is straightforward. From a 3-partite graph we build a *k*-partite graph by adding *k* − 3 new vertices, each new vertex adjacent to all others, and each constituting a new partite set. The 3-partite graph has a 3-partite clique of size *p* iff the *k*-partite graph has a *k*-partite clique of size (*p* + *k* − 3). □

Having thus settled the complexity of decision, we now return to search and optimization, and conclude from Theorem 3 that finding a vertex-maximum *k*-partite clique in a *k*-partite graph is *NP*-hard for all *k* ≥ 3.

## 5. A Special Class of Multipartite Graphs

We now consider and formalize a special class of *k*-partite graphs that can arise in a variety of classification contexts, from social tagging [30] to data alignment [31]. A good example arises in functional genomics. In this particular application, terms from multiple ontologies, each ontology represented as a partite set, are annotated to a common set of entities, in this case genes, which is then represented as a partite set of its own. Consider, for example, annotations from Gene Ontology [32] versus those from Mammalian Phenotype Ontology [33]. The former consists of relations between genes based on descriptive terminology about cellular location, biological process and/or molecular function. The latter maintains known relations between genes and the traits they influence. By examining overlaps common to these two ontologies, terms from one can be mapped onto terms of the other.

**Figure 6.**
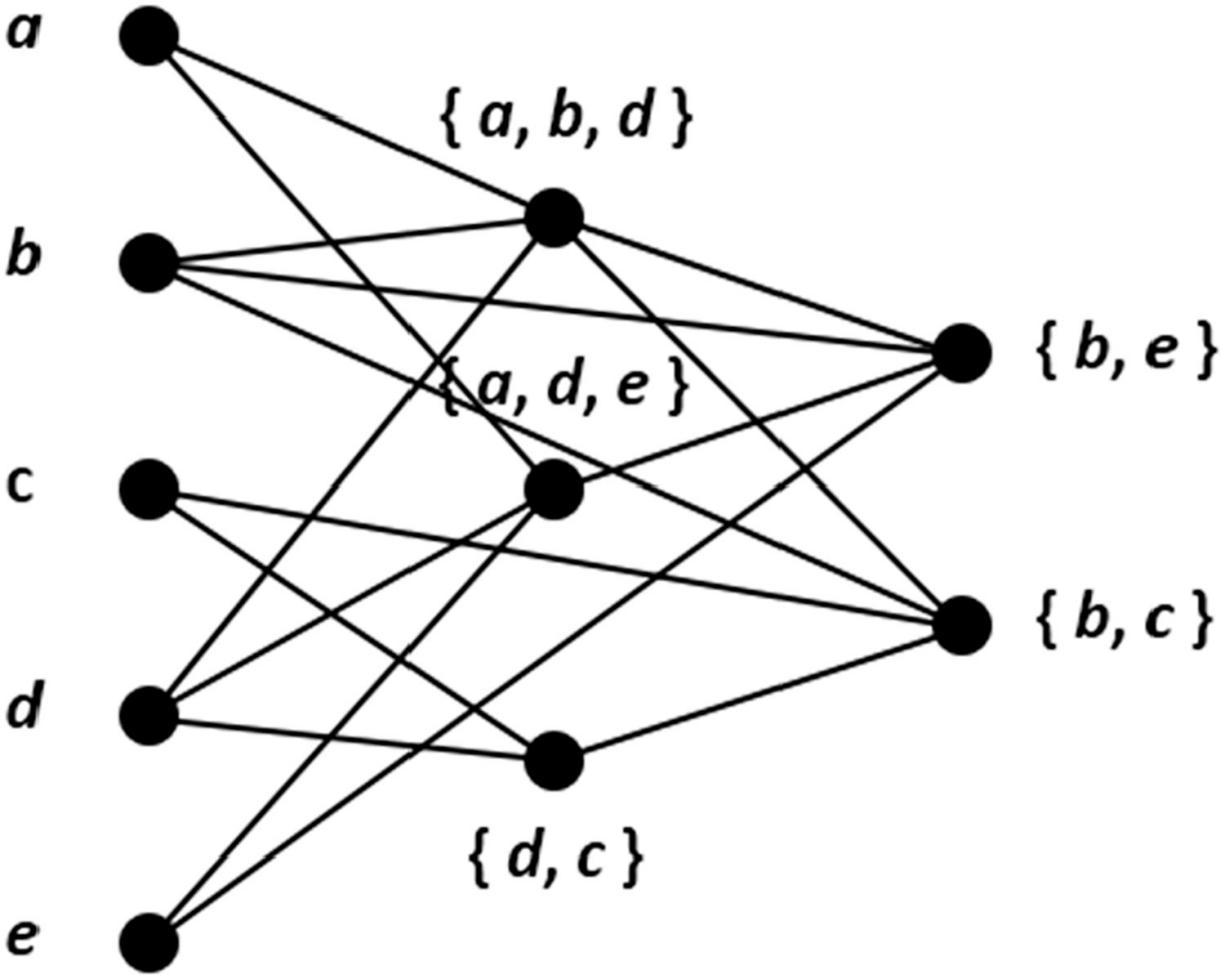
A 3-partite set intersection graph. Singleton vertices that represent *a*, *b*, *c*, *d*, and *e* comprise one partite set. Subset vertices that represent {*a*, *b*, *d*}, {*a*, *d*, *e*}, and {*d*, *c*} comprise a second partite set. A third partite set consists of subset vertices that represent {*b*, *e*} and {*b*, *c*}.

**Figure 7.**
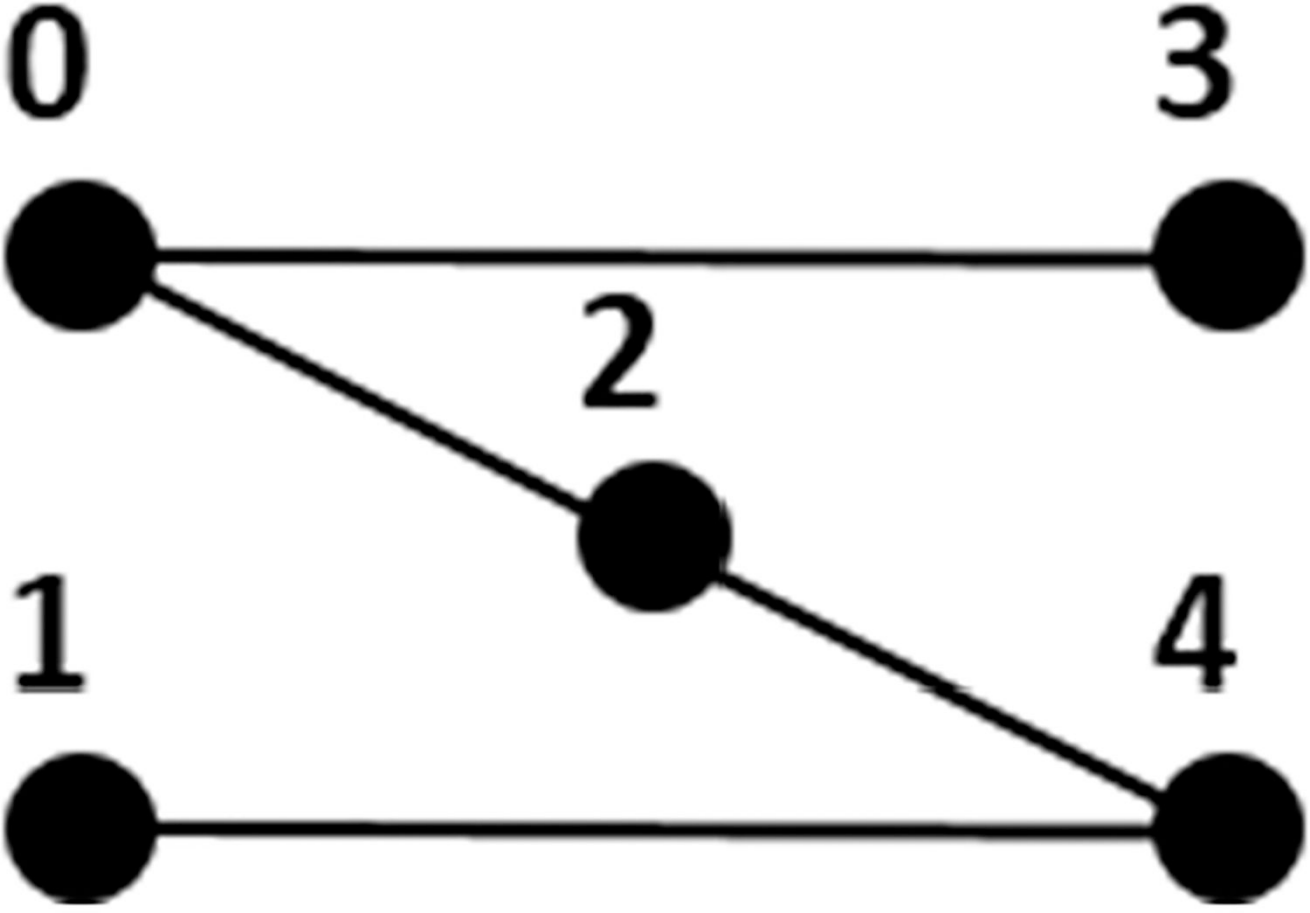
A 3-partite graph that is not a set intersection graph. Nodes labeled 0 and 1 in the leftmost partite set cannot both be singleton vertices, since otherwise nodes 2 and 3 must be adjacent. By the same token, nodes labeled 3 and 4 in the rightmost partite set cannot both be singleton vertices, else nodes 1 and 2 must be adjacent. But node 2 in the middle partite cannot be a singleton vertex either, because that would require nodes 0 and 4 to be adjacent.

To realize this class, let *S* denote a finite set. We define a *k*-partite set intersection graph to be a *k*-partite graph in which one partite set, say the first, contains *|S|* ‘singleton vertices’, one for each distinct element of *S*, and every other partite set contains some number of ‘subset vertices’ that represent (not necessarily distinct) nonempty subsets of *S*. A pair of vertices in different partite sets is joined by an edge iff the element or subset(s) they represent intersect. A sample 3-partite set intersection graph is shown in Figure 6, where vertices are labeled with the elements and subsets they represent. In contrast, a 3-partite graph that is not a 3-partite set intersection graph is depicted in Figure 7.

**Figure 8.**
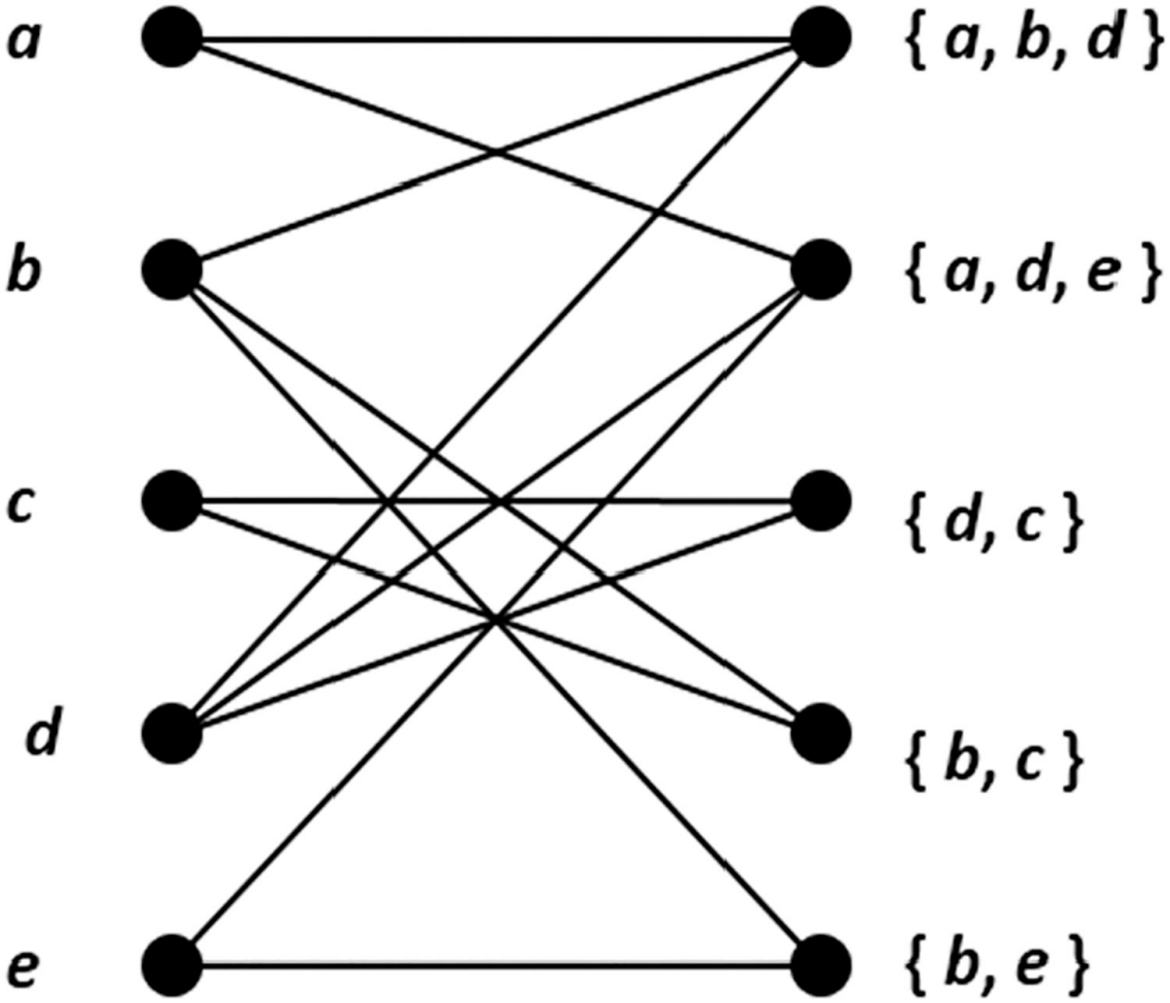
An example of graph compression. The *k*-partite set intersection graph of Figure 6 has been reduced to a bipartite set intersection graph.

We now show that the time required to enumerate maximal *k*-partite cliques can be reduced for *k*-partite set intersection graphs. Let *G* denote such a graph, with vertex set {*u*_1_, *u*_2_, …, *u*_*n*_}. To take advantage of *G*’s singleton structure, we compress it into a bipartite set intersection graph, *G*_*b*_, in which all subset vertices are fused into a single partite set. We denote the vertex set of *G*_*b*_ by {*v*_1_, *v*_2_, …, *v*_*n*_}, where *v*_*i*_ represents the same element or subset as does *u*_*i*_ for 1 ≤ *i* ≤ *n*. Interpartite edges in *G*_*b*_ are decided based on intersections, just as with *G*. Thus, the neighborhood (set of neighbors) of a singleton vertex is unaffected. This construction is illustrated in Figure 8.

As we shall prove shortly, we need only call MBEA [15] (currently the fastest published algorithm for enumerating maximal bicliques in bipartite graphs) on *G*_*b*_, then check each maximal biclique it produces to determine whether the corresponding vertices in *G* cover all partite sets. We call this algorithm MMCE-SI, for maximal multipartite clique enumeration on set intersection graphs, See Algorithm 2.

**Table:** 

Algorithm 2. MMCE-SI
1 input: a *k*-partite set intersection graph *G =* (*V*, *E*), with partite sets *V*_1_, *V*_2_, …, *V*_*k*_;
2 output: all maximal *k*-partite cliques in *G*;
3 compute the bipartite graph *G*_*b*_;
4 invoke MBEA on *G*_*b*_;
5 for each maximal biclique *B* returned by MBEA
6 if every partite set of *G* contains at least one *u*_*i*_ for which *v*_*i*_ ∈ *B*
7 then report {*u*_*i*_|*v*_*i*_ ∈ *B*} as a maximal *k*-partite clique;
8 end MMCE-SI

**Theorem 4.** *Let S, G, and G*_*b*_ *be defined as above, and let I denote an arbitrary subset of {1, 2*, … *, n}. If each partite set of G contains at least one member of U = {u*_*i*_*|i*∈ *I}, then U is a k-partite clique of G if V = {v*_*i*_*|i*∈ *I} is a biclique of G*_*b*_.

**Proof of Theorem 4.** Suppose *S*, *G*, *G*_*b*_, *I*, *U*, and *V* satisfy the statement of the theorem, and specifically that the vertices of *U* cover all partite sets of *G*. We first prove the forward (only if) implication, and suppose that *U* is a *k*-partite clique of *G*. Because singleton vertex neighborhoods are unchanged by compression, *V* must be a biclique of *G*_*b*_. We now prove the reverse (if) implication, and suppose *V* is a biclique of *G*_*b*_. We again use the fact that singleton vertex neighborhoods are unchanged by compression, and observe from this that we only need to examine the configuration in which *v*_*i*_ and *v*_*j*_ (*i,j* ∈ *I*) are subset vertices of *G*_*b*_, and *u*_*i*_ and *u*_*j*_ reside in different partite sets of *G*. Because *v*_*i*_ and *v*_*j*_ are in *V*, they have at least one singleton neighbor in common, and so the sets they represent cannot be disjoint. Therefore, *u*_*i*_ and *u*_*j*_ share an edge in *G*. It follows that *U* is a *k*-partite clique of *G*. □

It follows that maximality is preserved as well.

**Corollary 1.** *Let U and V be defined as in Theorem 4. Then U is a maximal k-partite clique of G if V is a maximal biclique of G*_*b*_.

Therefore, whenever *k* exceeds 2, enumerating maximal *k*-partite cliques in a *k*-partite set intersection graph can be accomplished by enumerating maximal bicliques in the manner just defined. We leave it as an exercise for the reader to construct counterexamples showing that *k*-partite graphs with edges based on intersections is insufficient by itself. One partite set must contain only singleton vertices for Theorem 4 to hold. Corollary 1 actually ensures that every maximal *k*-partite clique is matched by a (unique) maximal biclique. But the converse does not hold without partite set coverage. Consider, for example, the three vertices in Figures 6 and 8 that represent *b*, *c* and {b,c}. These form a maximal biclique in Figure 8, but not a maximal 3-partite clique in Figure 6.

**Corollary 2.** *The number of maximal k-partite cliques in a k-partite set intersection graph is bounded above by the number of maximal bicliques in its compressed bipartite graph*.

In addition to providing relative simplicity by dealing only with bipartite graphs, MMCE-SI offers tangible speedup over MMCE as well. To see this, note that the overall time needed by MMCE-SI is dominated by its call to MBEA. We know from [15] that MBEA takes at most *O*(|*E*|) time for each maximal biclique it reports, and from [20,22] that a bipartite graph can have at most *O*(2^*n*/2^) such bicliques. Because checking for partite set coverage can be accomplished in *O*(|*E*|) time, and because a bipartite graph can have no more than *n*^2^/4 edges, it follows that the total time required by MMCE-SI is *O*(*n*^2^2^*n*/2^), which is an asymptotic improvement over the *O*(3^*n*/3^) time complexity of MMCE.

As previously mentioned, it is *NP*-complete to decide whether a graph is *k*-partite. We have therefore assumed throughout that an input graph comes along with its *k*-partite structure. In this section, we have further assumed knowledge of supplemental information about *S* and how its elements and subsets relate to a *k*-partite graph’s vertices. Of course, *S* may not be unique. But what if it is not even known? In such an event, we first need to determine whether a *k*-partite graph is in fact a *k*-partite set intersection graph at all. Thus motivated, we now present a polynomial-time algorithm for this task, from which it follows that supplemental information about *S* is actually unneeded. We call this algorithm MSIGR, for multipartite set intersection graph recognition (see Algorithm 3), and base it on the following characterization.

**Theorem 5.** *Let G denote a k-partite graph. G is a k-partite set intersection graph if G contains some partite set P with the property that every pair of vertices in different partite sets other than P are either adjacent with a common neighbor in P, or nonadjacent with no common neighbor in P*.

**Proof of Theorem 5.** We first prove the forward (only if) implication. Let *G* denote an arbitrary *k*-partite set intersection graph. Then there exists some finite set *S* for which there is a partite set *P* containing only singleton vertices, one for each element of *S*. Let *u* and *v* denote a pair of vertices that reside in different partite sets, neither of which is *P*. If they are adjacent, then the subsets they represent share at least one element, which means they are adjacent to at least one common singleton vertex in *P* as well. On the other hand, if *u* and *v* are nonadjacent, then the subsets they represent are disjoint, and so they cannot be adjacent to a common singleton vertex in *P*.

We now prove the reverse (if) implication. Suppose we know only that *G* is a *k*-partite graph, with some partite set *P* satisfying the property stated in the theorem. Let *p* denote the cardinality of *P*, and let *S* denote the set {1, 2, …, *p*}. Associate with each vertex in *P* a unique element of *S*, and associate with each vertex not in *P* the subset of *S* represented by all its neighbors in *P*. Now suppose vertices *u* and *v* are adjacent. If either is in *P*, then the sets they represent intersect by construction. If neither is in *P*, then the fact that they must have a common neighbor in *P* means again that the sets they represent intersect. Conversely, suppose *u* and *v* are nonadjacent. If either is in *P*, then the sets they represent are disjoint by construction. And if neither is in *P*, then the fact that they must not have a common neighbor in *P* means again that the sets they represent are disjoint. *G* therefore satisfies the definition of a *k*-partite set intersection graph. □

**Table:** 

Algorithm 3. MSIGR
1 input: a *k*-partite graph *G* = (*V*,*E*), with partite sets *V*_1_, *V*_2_, …, *V*_*k*_;
2 ouput: “yes” or “no,” depending on whether *G* is a *k*-partite set intersection graph;
3 for each partite set *P* of *G*
4 *flag* ← *true*;
5 for every *u* and *v* in different partite sets, neither of which is *P*
6 if *u* and *v* are adjacent but have no common neighbor in *P*
7 then *flag* ← *false* and break for loop;
8 if *u* and *v* are nonadjacent but have a common neighbor in *P*
9 then *flag* ← *false* and break for loop;
10 if *flag* then report “yes” and halt;
11 report “no”;
12 end MSIGR

MSIGR’s outer loop makes at most *k* iterations, while its inner loop performs *O*(*n*^2^) checks, each check against *O*(*n*) neighbors. MSIGR therefore runs in *O*(*kn*^3^) time.

## 6. Summary and Directions for Future Research

With this work we have derived a number of new results concerning the enumeration of maximum and maximal *k*-partite cliques in *k*-partite graphs. In so doing, we have resolved fundamental questions concerning both asymptotic optimality and algorithmic complexity. We have modified the well-known Bron and Kerbosch approach so that it could be applied to multipartite graphs, and shown that the resultant algorithm runs in *O*(3^*n*/3^) time. We have proved that this matches the minimum asymptotic worst-case bound. We have also considered the problem of identifying a vertex-maximum *k*-partite clique and, unlike the bipartite case, shown it to be *NP*-hard for all *k* ≥ 3. We have introduced and studied *k*-partite set intersection graphs, proved them to be more efficiently solvable via a reduction to bipartite graphs, and developed an *O*(*kn*^3^) algorithm for their recognition.

Problems amenable to this general approach abound. A *k*-partite graph model may be reasonable whenever associations between heterogeneous data types can be scored with a similarity metric. In that regard, maximal *k*-partite cliques are a ‘gold standard’ for multidimensional density-based clustering. With minor algorithmic alterations, the range of MMCE’s applications can readily be widened. In a data mining setting, for example, a maximal *k*-partite clique corresponds to a maximal full space cluster. Checking that only *k*^0^ < *k* partite sets are covered (line 2 of ENUMERATE) produces an algorithm to enumerate all maximal subspace clusters of at least *k*^0^ dimensions. Similarly, MMCE can easily be modified to produce only vertex (edge) maximum *k*-partite cliques, or *k*-partite cliques with some pre-specified number vertices (edges).

I/O limitations are frequently overlooked or ignored, but in this particular case they merit some mention. Input is of course only an *O*(*n*^*2*^) operation. The standard output premise [9,10], however, and the one we have adopted here, is that *k*-partite cliques are merely reported, not written, as they are discovered. Because a clique may contain *O*(*n*) vertices, listing clique contents would add a linear factor to MMCE’s *O*(3^*n*/3^) run time. But we know from Theorem 2 that, for every fixed *k* ≥ 3, there are infinitely many *k*-partite graphs with ~3^*n*/3^ maximal *k*-partite cliques. Moreover, these cliques each contain *n*/3 vertices because of the way partite sets are tripartitioned. Any listing algorithm must therefore require Ω(*n*3^*n*/3^) time in the worst case, and so an adaptation of MMCE that outputs, not just reports, maximal cliques is also asymptotically optimal.

Numerous research questions beckon. For example, can heuristic strategies provide improvement? What if one were to start by removing any vertex not part of a 3-clique or without neighbors in every partite set? And what of pivot strategies? Are any selection techniques particularly well-suited for *k*-partite graphs? One might also entertain the idea of checking partite set coverage in more sophisticated ways, although an amortized form of analysis may be required. Finally, we consider the decision version of the edge-maximum biclique problem and observe that its proof of *NP*-completeness [24] resolves, perhaps unknowingly, a quadratic programming conjecture [34], which posited that minimizing a product of linear functions is *NP*-hard. The complexity-theoretic resolution of edge-maximum biclique actually settles a somewhat stronger version of the quadratic programming conjecture, one that restricts numeric variables to binary values. In contrast, the complexity of deciding edge-maximum *k*-partite clique for *k* ≥ 3 remains open. We are, quite naturally, confident that it too is *NP*-complete. Rather oddly, however, the proof for the bipartite case does not appear to generalize because of the sum-of-products formula used to determine the number of edges in a *k*-partite clique.

## Appendix A

We derive $\sum_{a=1}^{p-2}\sum_{b=1}^{p-a-1}\left(\begin{array}{c} p\\ a \end{array}\right)\left(\begin{array}{c} p-a\\ b \end{array}\right)=3^{n/3}-3\left(2^{n/3}-1\right)$, where *p* = *n*/3.

$$\sum_{a=1}^{p-2}\sum_{b=1}^{p-a-1}\left(\begin{array}{c} p\\ a \end{array}\right)\left(\begin{array}{c} p-a\\ b \end{array}\right)\;=\sum_{\text{a}=1}^{\text{p}-2}\left(\begin{array}{c} \text{P}\\ \text{a} \end{array}\right)\sum_{\text{b}=1}^{\text{p}-\text{a}-1}\left(\begin{array}{c} \text{p}-\text{a}\\ \text{b} \end{array}\right),\text{ which by }(*)\;=\sum_{\text{a}=1}^{\text{p}-2}\left(\begin{array}{l} \text{p}\\ \text{a} \end{array}\right)\left(2^{\text{p}-\text{a}}-2\right)\;=\sum_{\text{a}=0}^{\text{p}}\left(\begin{array}{c} \text{p}\\ \text{a} \end{array}\right)\left(2^{\text{p}-\text{a}}-2\right)-\left(2^{\text{p}}-2\right)+1\;=\sum_{\text{a}=0}^{\text{p}}\left[\left(\begin{array}{c} \text{p}\\ \text{a} \end{array}\right)\left(2^{\text{p}-\text{a}}\right)-2\left(\begin{array}{c} \text{p}\\ \text{a} \end{array}\right)\right]-\left(2^{\text{p}}-2\right)+1\;=\sum_{\text{a}=0}^{\text{p}}\left(\begin{array}{l} \text{p}\\ \text{a} \end{array}\right)\left(2^{\text{p}-\text{a}}\right)-\sum_{\text{a}=0}^{\text{p}}2\left(\begin{array}{l} \text{p}\\ \text{a} \end{array}\right)-\left(2^{\text{p}}-2\right)+1\;=\sum_{\text{a}=0}^{\text{p}}\left(\begin{array}{l} \text{p}\\ \text{a} \end{array}\right)\left(2^{\text{p}-\text{a}}\right)-2\left(2^{\text{p}}\right)-\left(2^{\text{p}}\right)+3,\text{ which by }(**)\;=\sum_{\text{a}=0}^{\text{p}}\left(\begin{array}{c} \text{P}\\ \text{a} \end{array}\right)\left(2^{\text{a}}\right)-2\left(2^{\text{p}}\right)-\left(2^{\text{p}}\right)+3,\text{ which by }(***)\;=3^{\text{p}}-2\left(2^{\text{p}}\right)-\left(2^{\text{p}}\right)+3\;=3^{\text{p}}-3\left(2^{\text{p}}-1\right)\;=3^{\text{n}/3}-3\left(2^{\text{n}/3}-1\right)$$

(*) Observe that $\sum_{k=1}^{n-1}\left(\begin{array}{l} n\\ k \end{array}\right)=2^{n}-2$.

(**) To see that $\sum_{a=0}^{p}\left(2^{p-a}\right)=\sum_{a=0}^{p}\left(2^{a}\right)$, we expand the summations

(A1) $$\sum_{a=0}^{p}\left(\begin{array}{c} p\\ a \end{array}\right)\left(2^{a}\right)=\left(\begin{array}{c} p\\ 0 \end{array}\right)\left(2^{0}\right)+\left(\begin{array}{c} p\\ 1 \end{array}\right)\left(2^{1}\right)+\ldots +\left(\begin{array}{c} p\\ p-1 \end{array}\right)\left(2^{p-1}\right)+\left(\begin{array}{c} p\\ p \end{array}\right)\left(2^{p}\right)$$

(A2) $$\sum_{a=0}^{p}\left(\begin{array}{c} p\\ a \end{array}\right)\left(2^{p-a}\right)=\left(\begin{array}{c} p\\ 0 \end{array}\right)\left(2^{p}\right)+\left(\begin{array}{c} p\\ 1 \end{array}\right)\left(2^{p-1}\right)+\ldots +\left(\begin{array}{c} p\\ p-1 \end{array}\right)\left(2^{1}\right)+\left(\begin{array}{c} p\\ p \end{array}\right)\left(2^{0}\right)$$

and exploit the symmetry given $\left(\begin{array}{l} n\\ k \end{array}\right)=\left(\begin{array}{c} n\\ n-k \end{array}\right)$ by. In Equation (A1) or (A2), we can substitute $\left(\begin{array}{c} p\\ p-p \end{array}\right)$ for $\left(\begin{array}{l} p\\ p \end{array}\right)$, $\left(\begin{array}{c} p\\ p-(p-1) \end{array}\right)$ for $\left(\begin{array}{c} p\\ p-1 \end{array}\right)$, $\left(\begin{array}{c} p\\ p-(p-2) \end{array}\right)$ for $\left(\begin{array}{c} p\\ p-2 \end{array}\right)$, …, and $\left(\begin{array}{c} p\\ p-(p-p) \end{array}\right)$ for $\left(\begin{array}{l} p\\ 0 \end{array}\right)$ to yield the other equation.

(***) By the binomial theorem, $\sum_{k=0}^{n}\left(\begin{array}{l} n\\ k \end{array}\right)\left(x^{k}\right)={\left(1+x\right)}^{n}$.

## Appendix B

We first prove that $\underset{n\to \infty }{\text{lim}}\frac{{\left(3^{n/12}-3\left(2^{n/12}-1\right)\right)}^{4}}{3^{n/3}}=1$.

$$\underset{n\to \infty }{\text{lim}}\frac{{\left(3^{n/12}-3\left(2^{n/12}-1\right)\right)}^{4}}{3^{n/3}}\;=\underset{n\to \infty }{\text{lim}}{\left(\frac{3^{n/12}-3\left(2^{n/12}-1\right)}{3^{n/12}}\right)}^{4}\;=\underset{n\to \infty }{\text{lim}}{\left(1-3\frac{2^{n/12}-1}{3^{n/12}}\right)}^{4}\;=\underset{n\to \infty }{\text{lim}}{\left(1-3\left({\left(\frac{2}{3}\right)}^{n/12}-\frac{1}{3^{n/12}}\right)\right)}^{4}\;={\left(\underset{n\to \infty }{\text{lim}}\left(1-3\left({\left(\frac{2}{3}\right)}^{n/12}-\frac{1}{3^{n/12}}\right)\right)\right)}^{4}\;={\left(1-3\left(\underset{n\to \infty }{\text{lim}}{\left(\frac{2}{3}\right)}^{n/12}-\underset{n\to \infty }{\text{lim}}\frac{1}{3^{n/12}}\right)\right)}^{4}\;={\left(1-3\left(0-0\right)\right)}^{4}=1^{4}=1$$

A similar series of arguments shows that $\underset{n\to \infty }{\text{lim}}\frac{{\left(3^{n/15}-3\left(2^{n/15}-1\right)\right)}^{5}}{3^{n/3}}=1$, and in general that $\underset{n\to \infty }{\text{lim}}\frac{{\left(3^{n/3k}-3\left(2^{n/3k}-1\right)\right)}^{k}}{3^{n/3}}=1$, for any *k* ≥ 6.

## Appendix C

We provide a counterexample to the claim in [28] that some fact in [35] guarantees that at least half the clauses in any one-in-three SAT instance can be simultaneously one-in-three satisfied. Consider the eight-clause Boolean expression (*a* ∨ *b* ∨ *c*) ∧ (*a* ∨ *b* ∨¬*c*) ∧ (*a* ∨¬*b* ∨ *c*) ∧ (*a* ∨¬*b* ∨¬*c*) ∧ (¬*a* ∨ *b* ∨ *c*) ∧ (¬*a* ∨ *b* ∨¬*c*) ∧ (¬*a* ∨¬*b* ∨ *c*) ∧ (¬*a* ∨¬*b* ∨¬*c*). It is elementary to verify that each of the eight possible true/false assignments to the elements of {*a*, *b*, *c*} simultaneously one-in-three satisfy exactly three clauses.
